# The myeloid switch: immune drivers in atopic dermatitis — roles in pathogenesis and emerging therapeutic targeting

**DOI:** 10.3389/fimmu.2025.1608338

**Published:** 2025-06-30

**Authors:** Erik Kupschke, Mirjam Schenk

**Affiliations:** ^1^ Christine Kühne – Center for Allergy Research and Education, Davos, Switzerland; ^2^ Graduate School for Cellular and Biomedical Sciences, University of Bern, Bern, Switzerland; ^3^ Institute of Tissue Medicine and Pathology, Experimental Pathology, University of Bern, Bern, Switzerland

**Keywords:** atopic dermatitis, myeloid cells, dendritic cells, immunology, inflammatory disease, therapeutic targets, immune drivers

## Abstract

Atopic dermatitis (AD) is one of the most common chronic inflammatory skin diseases worldwide, significantly impairing patients’ quality of life. It is characterized by recurrent eczematous lesions, intense pruritus, and disruption of the epidermal barrier. The pathogenesis of AD is multifactorial and involves complex interactions between genetic predisposition, environmental triggers, skin barrier defects, microbial dysbiosis, and immune dysregulation. While much of the research in recent decades has focused on the type 2 helper T cell (Th2)-driven adaptive immune responses that dominate the acute phase of the disease, the role of innate immunity—particularly that of myeloid cells—has emerged as a crucial and underrated component in disease pathogenesis and progression. This review highlights recent findings on the role of myeloid cells in the initiation, maintenance, and amplification of inflammation in AD. Myeloid cells respond to a wide range of environmental and tissue-derived triggers, including cytokines, alarmins, and microbial products. Upon activation, they contribute to the inflammatory milieu by producing chemokines and cytokines, presenting antigens, and recruiting other immune cells to the skin. Importantly, myeloid cells not only shape the local immune landscape but also engage in crosstalk with keratinocytes and adaptive immune cells, thereby reinforcing chronic inflammation. In addition, the review outlines emerging therapeutic strategies aimed at modulating myeloid cell function or selectively targeting pro-inflammatory subsets. These approaches offer promising avenues that complement existing Th2-centered therapies, addressing disease mechanisms beyond the adaptive immune response. A deeper understanding of the diverse and dynamic roles of myeloid cells in AD may thus support the development of more comprehensive and personalized treatment strategies for long-term disease control.

## Introduction to atopic dermatitis

Atopic dermatitis (AD) is one of the most common inflammatory skin diseases worldwide. It often starts in early childhood with onset before age of 5 years but can also develop in adults. AD is characterized by intense pruritus, dry skin, and recurrent eczematous lesions. The prevalence of AD has increased 2-3-fold in recent decades, currently affecting approximately 25% of children and 4-7% of adults in industrialized nations (1). This rising incidence has made AD a significant public health concern due to its substantial impact on quality of life and economic burden. The pathophysiology of AD is complex, involving genetic, immunologic, and environmental factors that lead to skin barrier dysfunction and immune dysregulation. Impaired skin barrier function increases susceptibility to irritants, allergens, and microbial colonization, particularly by *Staphylococcus aureus* (*S. aureus*) which can trigger inflammatory responses involving both innate and adaptive immunity. AD is often associated with other atopic conditions like asthma, allergic rhinitis, and food allergies as part of the “atopic march.” Management typically involves an integrated approach including skin care, anti-inflammatory treatments, and, in some cases, emerging targeted therapies (**Table 1**). Early diagnosis and intervention are important to control symptoms, prevent complications, and improve quality of life for patients with this challenging chronic condition. In the early stages of the disease, it is easier to break the itch-scratch cycle, as it worsens with each iteration. The inflammation causes itching, which leads to scratching. Scratching disrupts the skin barrier, allowing allergens to enter and triggering stronger inflammation. Therefore, early diagnosis and treatment are crucial to control symptoms, prevent complications, and improve the quality of life for patients with this challenging chronic condition.

**Table 1:** Current therapeutical approaches to treat atopic dermatitis, sorted by treatment strategy.

Treatment strategy	Target	Drug	Phase	Study ID
Adaptive immunity	AhR-Agonist	Tapinarof/Benvitimod	IIb	NA
	IgE	Ligelizumab	II	NCT01552629
		Omalizumab	IV	NCT00367016
			IV	NCT02300701
	IL-13	Tralokinumab	Approved in EU	NCT03526861
	IL-13Rα1	ASLAN004	Ib	NCT04090229
	IL-33	Astegolimab	IIa	NCT03747575
	IL-33	MEDI3506	IIa	NCT04212169
	IL-4Rα	AK120	Ib	NCT04256174
	OX40	GBR 830/ISB 830	IIb	NCT03568162
	TSLP	Tezepelumab	II	NCT03809663
			IIa	NCT02525094
Adaptive immunity Th17	IL-17A	Secukinumab	II	NCT03568136
			II	NCT02594098
Adaptive immunity Th2	IL-4	Pitakinra	II	NCT00676884
	IL-13	Lebrikizumab	III	NCT04146363 (ADvocate1)
			III	NCT04178967 (ADvocate2)
			III	NCT04250337 (Adhere)
			III	NCT04760314 (Adhere-J)
			III	NCT04250350 (Adore)
		Tralokinumab	III	NCT03160885 (ECZTRA 2)
			III	NCT03131648 (ECZTRA 1)
			III	NCT03363854 (ECZTRA 3)
			III	NCT03526861 (ECZTRA 6)
			III	NCT03587805 (ECZTEND)
			III	NCT03761537 (ECZTRA 7)
			III	NCT04587453
	IL-4Rα	CBP-201	II	NCT05017480
			IIb	NCT04444752
		CM310	III	NCT05265923
			III	NCT04893707
			II	NCT04805411
		Dupilumab	III	NCT02260986 (LIBERTY AD CHRONOS)
			III	NCT02277743 (LIBERTY AD SOLO 1)
			III	NCT02277769 (LIBERTY AD SOLO 2)
			III	NCT02755649
			Approved globally	NCT03346434
	IL-5	Benralizumab	II	NCT03563066
			II	NCT04605094
		Mepolizumab		NCT03055195
	OX40	GBR830	II	NCT02683928
		KHK4083	IIb	NCT03703102
	OX40L	Amlitelimab, KY1005	IIa	NCT03754309
			II	NCT05131477
Adaptive immunity Th22	IL-17C	MOR106	II	NCT03864627
	IL-22	Fezakinumab/ILV094	II	NCT01941537
Innate immunity IL-1 family	IL-1α	Bermekimab	II	NCT04021862
			IIa	NCT03496974
	IL-33	Astegolimab	IIa	NCT03747575
		Etokimab	IIa	NCT03533751
		Itepekimab, REGN3500	IIa	NCT03738423
			II	NCT03736967
		MEDI3506	IIa	NCT04212169
	IL-36R	Spesolimab	II	NCT04086121
			IIa	NCT03822832
AA	COX and LOX	DS107/DGLA (dihomo gamma linolenic acid)	II	NCT02211417
			II	NCT02864498
			II	NCT03817190
	CRTH2	OC000459	IIb	NCT02002208
		QAW039/Fevipiprant	IIa	NCT01785602
IL-23	IL-12/23p40	Ustekinumab	II	NCT01806662
			II	NCT01945086
	IL-23p19	Risankizumab	II	NCT03706040
JAK-STAT	JAK/SYK	Gusacitinib (ASN002)	II	NCT03531957 (RADIANT)
			II	NCT03654755
	JAK1	Abrocitinib	III	NCT03349060 (JADE-MONO1)
			III	NCT03575871 (JADE-MONO2)
			III	NCT03627767 (JADE REGIMEN)
			III	NCT03720470 (JADE COMPARE)
			III	NCT03796676 (JADE TEEN)
			III	NCT04345367
		Upadacitinib	III	NCT03568318
			III	NCT03569293 (Measure Up 1)
			III	NCT03607422 (Measure Up 2)
			III	NCT03661138 (Rising Up)
			III	NCT03738397 (Heads Up)
	JAK1/2	Ruxolitinib cream	III	NCT03745638 (TRuE AD1)
			III	NCT03745651 (TRuE AD2)
		Baricitinib	III	NCT03334396(BREEZE-AD1)
			III	NCT03334422(BREEZE-AD2)
			III	NCT03334435(BREEZE-AD3)
			III	NCT03428100(BREEZE-AD4)
			III	NCT03435081(BREEZE-AD5)
			III	NCT03559270(BREEZE-AD6)
			III	NCT03733301 (BREEZE-AD7)
	JAK1/2/3, TYK2	Delgocitinib	III	JapicCTI-173554
			II	NCT03725722
	JAK1/3	ATI-1777 solution	II	NCT04598269
		Ifidancitinib (ATI-502) solution	II	NCT03585296
		Tofacitinib	IIa	NCT02001181
	JAK3, TrkA	SNA-125		Phase I/II
Modulation of microbiome	Protonophore activity	ATx201/Niclosamid	II	NCT04339985
	Cell membrane enhancer	CLS-001/Omiganan	II	NCT02456480
	Microbiom	STMC-103H	Ib	NCT03819881
	Modulation of systemic inflammation	EDP1815	Ib	NCT03733353
	Nitric oxide donor	B244	IIb	NCT04490109
	Targeted microbiome transplant	ShA9 (NIAID)	IIa	NCT03151148
	Targeted microbiome transplant (TLR5 and TNFR activation)	FB-401	IIb	NCT04504279
Myeloid Cells	MRGPRX2/mast cells	Celastrol	preclinical	10.1155/2023/9049256
	ERK/mast cells	CKBA	preclinical	doi.org/10.1002/eji.202350374
	HMGB1/Macrophages	Naringenin	preclinical	10.1111/exd.2016.25.issue-5
	Macrophages	Dictamnine	preclinical	10.1248/bpb.b23-00436
	Macrophages	Periploca forrestii saponin	preclinical	10.1155/2020/4346367
	Macrophages/keratinocytes	Codium fragile	preclinical	doi.org/10.4014/jmb.2312.12002
	mast cells	Non-thermal plasma	preclinical	doi.org/10.1038/s41598-019-49938-9
	TLR2 dendritic cells	anti-inflammatory topical therapy	preclinical	doi.org/10.1111/all.15899
	TNFα, CXC-10, IL-12, and IL-1b/macrophages	Stellariae Radix	preclinical	10.1155/2020/4346367
	TNFα, CXC-10, IL-12, and IL-1b/macrophages	Stellariae Radix	preclinical	10.1002/tox.24145
PDE	PDE4	Apremilast	II	NCT00931242
			II	NCT01393158
			II	NCT02087943
			II	NCT03160248
PDE inhibitors	PDE4	Crisaborole 2% ointment	III	NCT02118766 (AD-301)
			III	NCT02118792 (AD-302)
			III	NCT03645057
			III	NCT04040192
			III	NCT04360187
			III	NCT04498403
		Difamilast/OPA-15406 ointment	III	NCT03908970
			III	NCT03911401
			III	NCT03961529
		DRM02 gel	II	NCT01993420
		E6005 ointment	II	NCT01461941
		Lotamilast/	II	NCT03394677
		Lotamilast/RVT-501/E6005 ointment	II	NCT02950922
		Roflumilast/ ARQ-151 cream	II	NCT00746382
			IIa	NCT01856764
Pruritus	IL-31	BMS-981164	I	NCT01614756
	H4R	ZPL-3893787/Adriforant	II	NCT02424253
	IL-31RA	Nemolizumab	III	NCT01986933
			III	NCT03985943
	NK1R	Serlopitant	II	NCT02975206 (ATOMIK)
	NK1R	VLY-686/tradipitant	II	NCT02004041
			II	NCT02651714
			III	NCT03568331 (EPIONE)
			III	NCT04140695 (EPIONE2)
	OPRK1	Difelikefalin	II	NCT04018027
T-cell recruitment	CCR4	RPT193	I	NCT04271514

AA, arachidonic acid; PDE, phosphodiesterase.

Myeloid cells, including monocytes, macrophages, dendritic cells (DCs), and granulocytes, are crucial components of the innate immune system and play essential roles in skin homeostasis and inflammation. In the context of AD, these cells exhibit several intriguing characteristics that warrant further investigation, like phenotypic and functional changes in response to the skin microbiome. Furthermore, myeloid cells demonstrate dynamic roles throughout the progression of AD. For instance, myeloid-derived suppressor cells (MDSCs) show increased numbers and suppressive function in early stages of the disease but decreased presence in later stages. This dynamic behavior suggests a complex and evolving role for myeloid cells in the pathogenesis of AD.

## Role of myeloid cells in AD

### Monocytes and macrophages

Macrophages in the skin can originate form distinct sources. Tissue-resident macrophages which originate from yolk-sac-derived erythro-myeloid progenitors during early embryonic phase and get renewed frequently (2). The other type are macrophages originating from circulating monocytes, which are recruited to the skin in response to chemotactic signals. In addition to macrophages, monocytes can differentiate into DCs, amplifying the inflammatory response. Monocytes contribute to the inflammatory cycle in AD through production of cytokines and reactive oxygen species, and by their differentiation into active macrophages that drive skin inflammation (3). These macrophages are central players in inflammation, becoming activated at the onset of the inflammatory process. This activation triggers the expression of many genes, enhancing their ability to eliminate bacteria and regulate other cells through cytokine and chemokine secretion (4). However, excessive macrophage activation can be detrimental, contributing to conditions such as septic shock, organ dysfunction syndrome, and chronic inflammatory diseases like psoriasis and AD (3, 5). Research by Kiekens et al. demonstrated that macrophage numbers are significantly increased in the skin of both the acute and chronic inflammation phase of AD compared to non-affected and healthy skin (6).

**Figure 1:**
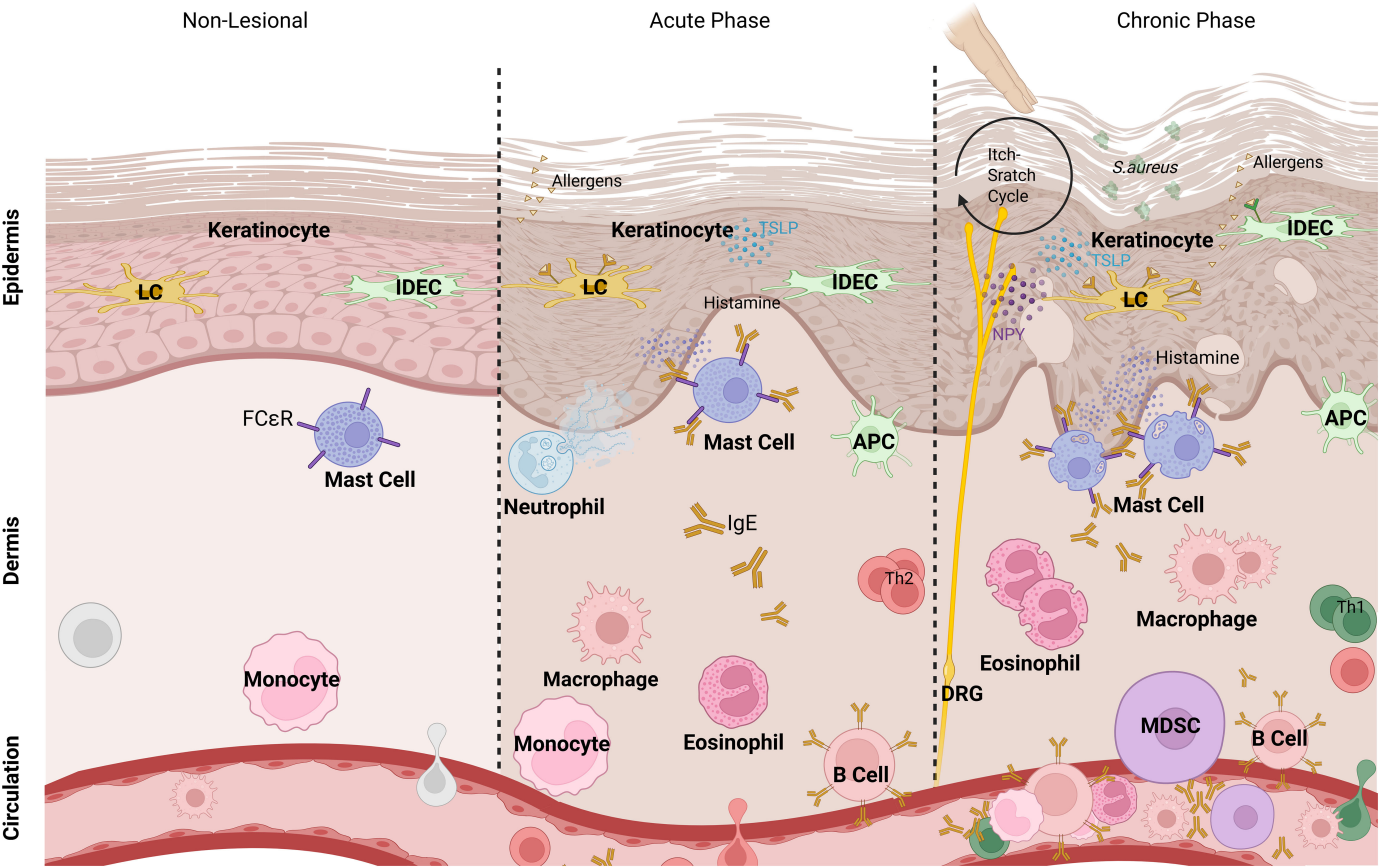
Myeloid cells in phases of AD. In acute and chronic phases of atopic dermatitis, keratinocytes are triggered by allergens that penetrate the disrupted skin barrier. Epidermal Langerhans cells (LCs), inflammatory dendritic epidermal cells (IDECs), and dermal macrophages capture antigens and become antigen-presenting cells (APCs), while additional immune cells like myeloid-derived suppressor cells (MDSCs) migrate to the inflamed area. Upon triggering, mast cells release histamine, while neutrophils and eosinophils release their granules. T cells, particularly Th2 cells in acute phase and Th1/Th17 cells in chronic phase, contribute to the inflammatory response. Dorsal root ganglia (DRG) interact with immune cells via neuropeptides, including neuropeptide Y (NPY), promoting itch sensation and further inflammation.

In acute and chronic AD lesions, the majority of macrophages originate from circulating monocytes that infiltrate the dermis, where they become key players in both immune defense and inflammation (**Figure 1**). Whereas they are relative scarce in non-lesional skin. These macrophages, function as antigen-presenting cells (APCs), play a role in antimicrobial activity, preventing bacterial invasion, and drive inflammation by releasing pro-inflammatory cytokines (e.g., TNF-α, IL-1β) and chemokines, which recruit additional immune cells (7). In contrast, tissue resident macrophages primarily maintain skin homeostasis and have regulatory functions during inflammation. Moreover, they participate in tissue remodeling and fibrosis by secreting matrix metalloproteinases (MMPs) and other related factors. Toll-like receptor stimuli and IFN-g polarize monocyte-derived macrophages toward the classical M1 activation pathway, resulting in production of high levels of proinflammatory cytokines (including IL-1, TNF-α, IL-12, and IL-23), reactive nitrogen, and oxygen radicals, enhanced microbicidal activity, and promotion of Th1 responses. Signal transducer and activator of transcription (STAT) 1 activation is crucial in this context. Phenotypically, M1 cells express high levels of MHC class II and co-stimulatory molecules, such as CD80 and CD86, and upregulate the expression of intracellular suppressor of cytokine signaling 3 (SOCS3) and inducible nitric oxide synthase (iNOS). Therefore, M1 cells are implicated in initiating and sustaining an inflammatory response (8).

Beyond their inflammatory functions, monocyte-derived macrophages in AD can undergo alternative activation, which is marked by the increased presence of CD163^+^ cells in lesional AD skin (9). These CD163^+^ macrophages, which are indicative of alternatively activated macrophages, are more abundant in lesional AD skin compared to healthy skin, and they share a similar distribution pattern with CD68^+^ cells. The alternative activation indicates a distinct role of these macrophages in the chronic inflammatory environment of AD, where they may exert several functions that support the persistent nature of the disease.

While the M1/M2 framework has long served as a useful model for understanding macrophage function, it is now recognized as an oversimplification, particularly in chronic inflammatory diseases such as atopic dermatitis. Macrophages exhibit considerable phenotypic and functional plasticity, continuously adapting to their microenvironment. In the skin of AD patients, macrophages are exposed to a complex array of stimuli, including type 2 cytokines (e.g., IL-4, IL-13), microbial components (e.g., *S. aureus*-derived peptidoglycan), neuropeptides (e.g., neuropeptide Y), histamine, lipid mediators, and mechanical stress, which shape their activation states dynamically (10). Among microbial stimuli, *S. aureus* is a predominant source of PAMPs in AD (11, 12). Its colonization exacerbates barrier dysfunction and drives macrophage activation through TLR2/6 signaling, inducing proinflammatory cytokines such as IL-1β, TNF-α, and IL-6. These cytokines contribute to the inflammatory milieu and promote the differentiation of Th22s and Th17 cells. Consistently, elevated levels of Th2 and Th22 cytokines are observed in both acute and chronic AD lesions, where they further impair barrier integrity and may contribute to microbial dysbiosis (13). Prolonged exposure to *S. aureus* can also functionally reprogram macrophages, promoting regulatory or tolerogenic phenotypes beyond classical polarization states. Recent single-cell transcriptomic studies have identified macrophage subsets in inflamed human skin that defy traditional M1/M2 classification, instead displaying hybrid or intermediate profiles (14, 15). These include cells co-expressing pro- and anti-inflammatory markers or those with specific metabolic or tissue-remodeling programs. Such macrophage states may be further modulated by epigenetic reprogramming, tolerization due to chronic microbial exposure, or neuroimmune feedback. In AD skin, for instance, CD68^+^CD163^+^ cells co-expressing DC markers like CD1a suggest the presence of a heterogeneous pool of monocyte-derived macrophages with dual immunomodulatory and antigen-presenting functions (6). Understanding this spectrum of macrophage activation is critical for deciphering their roles in disease progression and resolution. It also holds therapeutic potential, as interventions may target distinct macrophage subtypes or reprogramming pathways, such as through TRPV4 activation or cytokine modulation, rather than broadly inhibiting or activating these cells.

Additionally, Chitinase 3-like 1 (CHI3L1), a recognized mediator in Th2-driven inflammation which is also known as breast regression protein 39 (BRP-39), has been shown to mediate the development of AD through the activation of macrophages (16). M2 macrophages, a subtype often associated with anti-inflammatory responses, have been shown to reduce disease severity in a mouse model of AD when selectively activated through the mannose receptor CD206 using the bee venom component phospholipase A2 (17). On the contrary, they have been identified as a major source of IL-31 in AD by the immunohistochemical analysis of skin biopsy samples from AD patients. Their interactions with TSLP, periostin, and basophils further contribute to AD pathogenesis and the perpetuation of the itch-scratch cycle (18). M2 macrophages are also known to produce C–C motif chemokine ligand 18 (CCL18), a chemokine strongly associated with increased morbidity in AD patients (19). IL-4 and IL-13 significantly upregulate CCL18 expression, with IL-10 also contributing, but to a lesser extent. Histamine further enhances the cytokine-induced upregulation of CCL18 mRNA expression by stimulating the histamine receptor 2 (H2R), with the strongest effect observed in IL-10-stimulated macrophages. These combined activations in macrophages drive a substantial increase in CCL18 expression, resulting in its notably high levels in lesional AD skin and in serum of affected individuals. IL-4 upregulates both H2R and H4R, while IL-13 exclusively upregulates H4R without affecting H2R. Conversely, IL-10 upregulates H2R expression but shows a trend toward downregulating H4R (20). Moreover, recent *in vitro* studies have identified that the transient receptor potential vanilloid 4 (TRPV4) in macrophages, exerts anti-inflammatory properties. Its activation leads to the suppression of IL-1β expression in human macrophages by inhibiting NF-κB signaling and further prevents the differentiation of monocytes into pro-inflammatory macrophages, suggesting a potential therapeutic target for modulating macrophage activity in AD (21). Such findings align with the emerging understanding that macrophage functions in AD are shaped not only by cytokine milieu but also by mechanical and metabolic cues, including ion channels, microbial ligands, and tissue stressors.

In inflamed AD lesions, macrophage markers such as RFD7, which identifies mature tissue phagocytes, and CD68 exhibit similar expression levels and distribution patterns, with CD68^+^ macrophages being more prevalent than RFD7^+^ macrophages. Macrophages with high CD36 expression are also more abundant in inflamed AD lesions. CD36, a membrane glycoprotein involved in the phagocytosis of apoptotic cells such as neutrophils, plays a crucial role in limiting tissue damage and contributing to the resolution of inflammation in AD (22–24). In addition, in inflamed AD skin macrophages and DCs share overlapping phenotypes. Both DCs and macrophages express mannose receptors (MRs) for efficient antigen uptake, with MR expression predominantly found in monocyte-derived macrophages in inflamed AD skin (25). Kiekens et al. demonstrated that some macrophage populations may express macrophage (e.g., CD68) and DC (e.g., CD1a) markers simultaneously, indicating a heterogeneous pool of macrophage/DC-like cells (6). This overlap suggests complex plasticity of macrophages and DCs in AD, contributing to disease pathology.

### Dendritic cells

**Figure 2:**
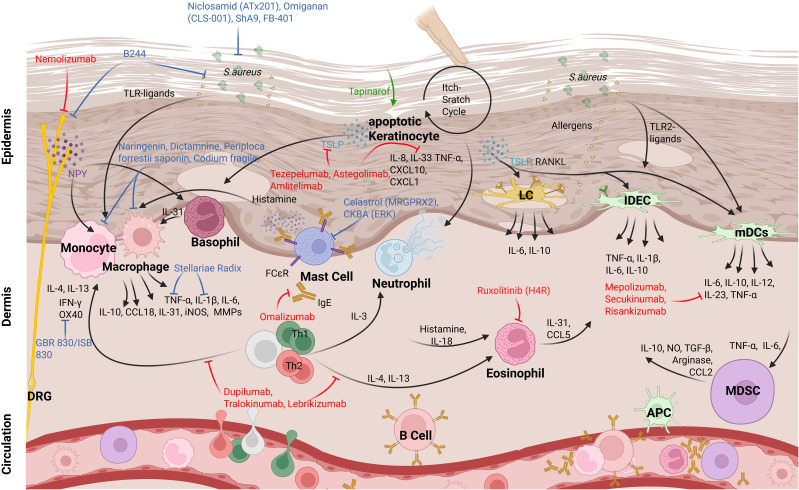
Myeloid cell interactions and therapeutic targets in AD skin. In AD skin, myeloid cells—including Langerhans cells (LCs), inflammatory dendritic epidermal cells (IDECs), monocyte-derived dendritic cells (mDCs), myeloid-derived suppressor cells (MDSCs), macrophages, and monocytes—interact with each other and with other immune and non-immune cells. These interactions are triggered by external stimuli (e.g., S. aureus compounds) and internal signals (e.g., TSLP from keratinocytes), leading to cytokine production (e.g., IL-6, IL-10, IL-4, IL-13). T cells are major drivers of inflammation. Current therapies (shown in red) and investigational treatments (in blue) mainly act by modulating cytokine pathways rather than directly targeting DC subsets. Some novel approaches, such as B244, target the skin microbiome to influence myeloid cell function.

DCs are key antigen-presenting cells that form a crucial link between innate and adaptive immunity, playing a significant role in the pathogenesis of AD. In AD patients, the numbers of specific myeloid Dendritic Cell (mDC) subsets, including Langerhans cells (LCs) in the epidermis and inflammatory dendritic epidermal cells (IDECs), are elevated in lesional skin (**Figure 1**). Activation of Toll-like receptors (TLRs) triggers the maturation of DCs, marked by the upregulation of costimulatory molecules and the release of cytokines (26) (**Figure 2**). Research on DCs in AD skin primarily focuses on TLR2, as it recognizes PAMPs from *S. aureus* (**Figure 1**), particularly peptidoglycan, a major component of its cell wall. DCs capture antigens and migrate to lymph nodes, where they present the antigens to naive T cells, thereby driving the differentiation of Th1 and Th2 cells. Th2 cells, which dominate the immune response during acute phases of AD, produce cytokines such as IL-4, IL-5, and IL-13 upon arrival in the inflamed skin, driving the allergic inflammation characteristic of AD. DCs are uniquely specialized to detect antigens from both external and internal sources, responding to various stress signals. Upon stimulation, DCs initiate and regulate adaptive immunity, performing a highly adaptable function that allows them to adjust their behavior to the specific tissue microenvironment, whether in the skin, lung, or gut mucosa. For example, DCs in the skin, such as LCs and IDECs, exhibit distinct marker expression, cytokine production, migration patterns, and metabolic adaptations compared to DC subsets like conventional DCs (cDCs) or plasmacytoid DCs (pDCs) in the lung or gut mucosa. Despite these dynamic and specialized roles in AD, research indicates that the absolute number of DCs remains unchanged in lesional compared to non-lesional AD skin (6, 27–30). In lesional AD skin, the marked dysbiosis of the microbiome, particularly the overgrowth of *S. aureus*, plays a critical role in shaping DC function. *S. aureus*-derived components such as peptidoglycan (PGN), lipoteichoic acid (LTA), and enterotoxins activate pattern recognition receptors (especially TLR2 and TLR6) on DCs, leading to the production of IL-6, IL-1β, and IL-23 (**Figure 2**). This stimulation promotes Th17 and Th2 polarization in a context-dependent manner. However, chronic microbial exposure may lead to DC tolerance or functional exhaustion, characterized by diminished cytokine production and impaired antigen presentation. This paradox, enhanced stimulation coupled with reduced functional responsiveness, highlights a hallmark of the dysregulated immune environment in chronic AD.

#### Langerhans cells

LCs are the main specialized subset of DCs located in normal epidermis, acting as sentinels of the immune system (**Figure 1**). They play a crucial role in initiating antigen-specific immune responses by efficiently taking up antigens in the skin, processing them, and presenting antigenic peptides to T cells in the draining lymph nodes. LCs are characterized by specific surface markers, including CD1a and CD207 (31). In addition to their well-established role in triggering specific immune responses through antigen presentation, LCs also exhibit regulatory functions. This regulatory capacity is partly mediated through the production of IL-10 or the activation of the aryl hydrocarbon receptor (32, 33). These functions may help explain findings by Igyarto et al., who observed that LC-deficient mice were more prone to contact hypersensitivity reactions compared to control mice (33). Further research has validated these regulatory functions in a skin graft mouse model, demonstrating that receptor activator of nuclear factor κB (RANK) is stimulated by its ligand (RANKL), which is produced by apoptotic keratinocytes, inducing LCs to produce IL-10 (34). This IL-10, in turn, induced the development of CD4^+^CD25^+^ Tregs, which can suppress skin immune responses (35). Kaplan et al. suggested that the presence of LC-derived IL-10 during the priming phase of the immune response may skew the T cell response toward a Th2 phenotype rather than a Th1 phenotype, potentially promoting the differentiation of Tregs (36). The Th2-dominant microenvironment in AD also contributes to reduced TLR2 expression on LCs. Th2 cytokines, such as IL-4 and IL-13, downregulate TLR2 expression, impairing the cell’s ability to respond to bacterial ligands and shifting the immune response away from Th1-mediated defense (37, 38). The Dysfunctional TLR2 signaling on LCs reduces cytokine production, including IL-6, which drives Th17 immune responses via the NF-κB pathway, and IL-10. While no direct link between TLR2 and IL-10 is known, its downregulation has been observed, suggesting altered immune regulation. In acute AD, these cytokines are impaired, diminishing the skin’s ability to control inflammation and bacterial infections (38–40). In healthy skin, LCs respond to bacterial signals such as *S. aureus* by maturing and migrating in response to TLR2 ligation, which is essential for initiating an effective immune defense. However, in AD skin, freshly isolated LCs show significantly lower TLR2 expression compared to keratinocytes and their healthy counterparts (41, 42). This low expression prevents LCs from maturing and migrating properly when stimulated with Pam3Cys, a synthetic TLR1/2 ligand that mimics *S. aureus* signals by replicating the structure of its lipoprotein component (43, 44). As a result, LCs in AD fail to initiate an adequate immune response, which is crucial for fighting skin infections and regulating inflammation. This impairment is reflected by a reduction in antimicrobial peptides (AMPs) such as LL-37, HBD-2, and HBD-3 in AD skin, further compromising the skin’s ability to combat microbial colonization and infection, particularly by *S.aureus* (45, 46). The impaired TLR2 response in LCs is not solely due to reduced receptor expression but also involves desensitization and tolerance, likely driven by chronic exposure to microbial ligands in AD skin. Prolonged colonization by *S. aureus* results in persistent TLR2 stimulation, contributing to immune cell desensitization, as observed in other immune cells such as monocytes and macrophages (45, 47). Desensitized immune cells fail to respond effectively to foreign PAMPs, potentially leading to a compromised immune response against subsequent infections.

#### Inflammatory dendritic epidermal cells

IDECs are pivotal in the pathophysiology of AD. This FcϵRI-positive subtype of mDCs contribute significantly to the inflammatory milieu and maintenance of the inflammatory reaction in AD by producing high levels of proinflammatory cytokines (48). Since LCs are more associated with a local Th2 response in acute AD lesions, IDECs contribute to the transition from a Th2-dominated immune response to a more multifaced immune profile in chronic phases, involving Th1, Th2, and Th17, thereby shaping the overall immune landscape in the skin (**Figure 1**) (1, 49, 50). Unlike LCs, IDECs are highly matured in the ‘steady state’ in AD skin, yet they still exhibit a reduced TLR2 expression, similar to LCs (24). Despite this maturation, IDECs in AD skin fail to respond to TLR2 ligation, as evidenced by their inability to upregulate MHC class II, CD83 and the costimulatory molecules CD80, and CD86 after stimulation with Pam3Cys (25). This characteristics of IDECs in AD lesions further contribute to the impaired immune response and decreased production of IL-10 seen in AD.

IDECs are also involved in the sensitization process to environmental allergens, capturing these allergens that penetrate the epidermis and trigger IgE-mediated immune responses. The functional behavior of IDECs is broadly influenced by the surrounding inflammatory microenvironment, where locally released cytokines from keratinocytes and other immune cells can modulate their activity (51). In AD IDECs, much like LCs, seem to be desensitized to TLR2 signals due to chronic exposure to microbial ligands in AD skin, particularly from *S. aureus*. This prolonged exposure leads to tolerance, preventing proper activation and cytokine production by IDECs (42, 47). In healthy skin, IDECs, along with LCs, help control bacterial infections by recognizing and responding to microbial patterns through TLR2, but in AD, this mechanism is severely compromised. This desensitization likely stems from the altered skin microbiome in AD, where *S. aureus* colonization increases while *Staphylococcus epidermidis* fails to control its growth (52–54). This shift in microbial composition further contributes to the impaired TLR2 response, as described for LCs. Furthermore, IDECs are implicated in self-sensitization mechanisms, responding to keratinocyte-derived proteins released due to skin damage, which can induce a Th2 response to self-structures resembling environmental allergens. This Th2 dominated cytokine environment in AD downregulates TLR2 expression and skew IDEC function toward promoting allergic inflammation rather than fighting infection (37, 38, 55). Moreover, IDECs, like LCs, fail to produce sufficient IL-6 and IL-10 after TLR2 stimulation, which impairs their ability to control inflammation and regulate immune responses effectively (47, 56). This compromised function of IDECs further exacerbates the immune imbalance in AD, promoting persistent inflammation and heightened susceptibility to bacterial infections. Understanding the role of IDECs in AD not only sheds light on the mechanisms underlying this condition but also opens potential therapeutic avenues aimed at modulating their proinflammatory properties to enhance tolerance and mitigate inflammation in affected individuals.

### Myeloid-derived suppressor cells

MDSCs have long been studied for their crucial role in malignancy and tumor maintenance through immunosuppression. However, they are now increasingly recognized as significant players in inflammation. Originating from immature myeloid cells, MDSCs possess immunoregulatory functions and have the potential to differentiate into mature DCs, macrophages, or granulocytes. These cells are categorized into two subtypes: monocytic MDSCs, characterized by a CD14^+^, CD11b^+^, Ly6C^+^, and Ly6G^-^ phenotype, and polymorphonuclear MDSCs, identified by a CD14^-^, CD11b^+^, Ly6C^low^, and Ly6G^+^ phenotype (57, 58). MDSCs exert their immunosuppressive effects through direct cell-to-cell contact and by secreting interleukins and chemokines. Their suppression of T-cell activity is mediated by arginase and iNOS, both of which deplete L-arginine, a molecule essential for T-cell differentiation and proliferation (59) (**Figure 2**). The depletion of L-arginine results in the downregulation of CD3ζ and MHC class II, along with the inhibition of Janus kinase 3 (JAK3) and STAT5 in T cells, ultimately reducing T-cell proliferation (60). Additionally, iNOS produces nitric oxide (NO), which induces dose-dependent apoptosis in T cells by modulating Bcl-2 expression (61). The role of NO in cutaneous inflammation is closely related to its surrounding conditions and concentration, with both pro-inflammatory and anti-inflammatory effects reported (61). In a mouse model of AD, exposure to *S. aureus* activated TLR 2–6 in the skin, leading to IL-6 production by skin cells. This IL-6 subsequently stimulated the recruitment of suppressive CD11b^+^Gr1^+^ MDSCs, which inhibited T cell-mediated responses through the iNOS-dependent pathway (62). Ligation of TLR1–2 was observed to not stimulate MDSCs recruitment. MDSCs also induce Tregs response through CTLA4 and membrane-bound TGF-β, which suppresses natural killer cell cytotoxicity, as demonstrated in recent mouse studies (63, 64). Furthermore, MDSCs produce IL-10, which promotes the differentiation of macrophages into predominantly anti-inflammatory M2 cells and fosters a Th2-mediated immune response (65, 66).

The therapeutic potential of MDSCs has been demonstrated in several mouse models, where these cells were attracted to inflamed sites through chemotactic stimuli. MDSCs were observed migrating to the spleen, lungs, lymph nodes, and inflamed skin, suggesting they selectively infiltrate inflamed tissues (67–73). However, infiltration into the skin was observed only in AD mice, reinforcing the idea that MDSCs selectively target sites of inflammation (72, 73). In one study, this was achieved by inducing CCR5 expression, while another study utilized the injection of CXCL17 (74, 75). Furthermore, MDSCs generated from human umbilical cord blood (hUCB) increased IFN-γ expression in the spleen and lymph nodes. IFN-γ is known for its therapeutic effects in AD (76, 77) and its potential to enhance the immunomodulatory capabilities of MDSCs (78, 79). However, it is important to note that mouse-derived IFN-γ cannot bind to the human IFN-γ receptor, rendering it incapable of affecting human MDSCs (80). Therefore, further studies are necessary to elucidate the role of IFN-γ induced by MDSCs in AD mouse models.

It is generally accepted that the numbers of immature granulocytic and monocytic MDSCs are elevated in patients with inflammatory conditions. This observation has been consistently reported in patients with inflammatory skin diseases and inflammatory bowel disease (58, 81–83). hUCB-MDSCs injected into mice with AD-like symptoms induced by *Dermatophagoides farinae* (Df) alleviated skin lesions in a dose-dependent manner, with higher doses (1 × 10^5^; and 1 × 10^6^ cells) proving more effective than lower doses (1 × 10^4^ cells). The MDSCs also reduced epidermal thickness and decreased inflammatory cell infiltration (11, 64). MDSCs (1 × 10^5^ and/or 1 × 10^6^ cells) restored skin barrier function and improved skin fibrosis, suggesting that the anti-inflammatory effects and wound-healing capacities of MDSC therapy contribute to recovery from skin barrier impairment, dysfunction, and skin fibrosis in Df-induced AD-NC/Nga mice. This likely results from abnormal repair in response to skin damage. Additionally, MDSC treatment reduced IgE production and lowered Th2- and Th17-mediated cytokine levels (64, 84).

Contrasting with findings in AD, MDSCs from psoriasis patients may recruit Tregs, which are less capable of exerting their regulatory functions (82, 85). They also may exhibit reduced expression of surface PD-1 and lower production of the anti-inflammatory cytokine IL-10 (58, 85). To becoming functionally deficient, MDSCs can acquire proinflammatory functions when exposed to an inflammatory environment, especially in psoriasis patients. These proinflammatory functions include the overexpression of IL-1β, IL-6, IL-8, and TNF-α (86, 87); the production of MMPs such as MMP1 and MMP9, which facilitate the transmigration and accumulation of MDSCs in tissues (88); and the secretion of monocyte chemoattractant protein 1 (MCP1), which acts as a chemoattractant for proinflammatory cells (82). Additionally, GRO and IL-8 secreted by MDSCs recruit neutrophils to the skin and inflammatory cells like neutrophils and monocytes to organs beyond the skin (89).

### Granulocytes

Granulocytes including neutrophils, eosinophils, and basophils, play critical roles in the pathogenesis of AD. Basophils contribute to the initiation of AD by increasing IL-4 expression and interacting with keratinocytes and dermal macrophages, leading to epidermal hyperplasia and skin barrier dysfunction, but play a minor role in chronic lesions (90, 91). It was observed that by stimulation with TSLP basophils interact with neutrophils, sensory neurons and T cells enhancing the inflammation and itch in AD skin (90). The interaction of basophils and neutrophils contributes to the severity of AD whereas the reduction of basophils leads to decreased infiltration of eosinophils and neutrophils, as well as skin thickness (92).

#### Eosinophils

Eosinophils in AD patients show increased and upregulated expression of histamine receptor 4 (H4R), driven by IL-4 and IL-13 through the JAK/STAT pathway, which results in elevated IL-31 production (93). The use of a direct H4R antagonist has been shown to improve disease severity, primarily by targeting pruritus (94). Moreover, the IL-18 receptor (IL-18Rα) is upregulated in eosinophils of AD patients, with histamine enhancing IL-18 expression through H2R and H4R, highlighting the roles of IL-18 and histamine in eosinophil-mediated inflammation in AD (95). Notably, the JAK-inhibitor Ruxolitinib significantly reduces H4R expression in eosinophils (**Figure 2**), presenting a treatment for AD (93).

#### Neutrophils

Neutrophils are involved in the inflammatory processes of AD particularly during acute phases or when secondary infections occur (**Figure 1**). They infiltrate the skin and contribute to inflammation, although their presence is not as prominent as in other skin conditions like psoriasis (96). But when they are increased in the lesional skin, the number is comparable to the number of neutrophils in psoriasis skin (97). In irritated skin the rapid infiltration of neutrophils was observed recently especially in Asian forms of AD (98, 99). This mobilization of neutrophils is affected by the Th2 cytokine mediated Th2-STAT6-C3 complement-NETs (neutrophil extracellular traps) cascade (100). The neutrophil-to-lymphocyte ratio (NLR), a serum inflammatory parameter, is a prognostic factor in several diseases (101–103). The NLR level in AD patients was observed to be higher than in healthy individuals and correlates with the severity of the inflamed and lesional skin (104–107). Therefore, elevated circulating neutrophils are more and more associated with AD severity, and a high NLR may serve as a parameter for AD severity.

TNFα released by mast cells can directly prime circulating neutrophils and enables them to migrate to surrounding tissue (108). Together with the dominant colonization of *S. aureus*, mast cells are the main trigger for neutrophil host response against microbial infections like NETs (109). Thus, NET formation is regulated by mast cell tryptase *in vivo (* 110). Next to TNFα several cytokines like IL-3, IL-8, and IL-33 affect neutrophils on a genetic or functional level (111–113) (**Figure 2**). In recent studies it was found that the elevated serum level of high-mobility group box 1 protein (HMGB1) correlates with AD severity in patients (114). HMGB1 promotes the attraction of neutrophils to skin wounds and supports the development of NETs. Additionally, extracellular HMGB1 can contribute to tissue damage by inducing NET formation under inflammatory conditions (115, 116).

As mentioned before, neutrophils infiltrate the tissue in early stages during the development of AD lesions. The recruitment is mediated through CXCR3 signaling, which initially gets activated by inflammatory cytokines such as CXCL1 and CXCL10 (117). CXCR3 activated neutrophils additionally start producing cytokines which amplify the inflammatory response. The chemokine IL-8 (CXCL8), detected in the lesional stratum corneum of AD patients, is linked to skin-barrier dysfunction. By binding to CXCR1 and CXCR2 receptors on human neutrophils, IL-8 activates various signaling pathways (118).

AD is characterized by a complex interplay between myeloid cells and other immune cells, with significant contributions from various cytokines and receptors that drive inflammation and pruritus. The Th2 cytokines, IL-4, IL-13, and IL-31, are key players in this process. These cytokines activate sensory fibers by engaging the TRP channel ankyrin transmembrane protein 1 (TRPA1) and the transient receptor potential (TRP) channel vallinoid 1 (TRPV1), which facilitate calcium influx into these fibers, and amplifies the sensation of itch (119). TRP channels, which respond to a variety of signals including chemical compounds, mechanical stimuli, temperature changes, and osmotic stress, are crucial not only in sensory perception but also in the progression of AD. Notably, the activation of TRPV3 on T cells can further increase TSLP production and the sensation of itch (120). TSLP, secreted by keratinocytes, interacts directly with sensory neurons through its receptor, further contributing to pruritus. The release of TSLP by epidermal cells is enhanced by activated T cells and through crosstalk with other immune cells, highlighting its central role in AD pathogenesis (121). The clinical efficacy of targeting these pathways is underscored by the use of anti-TSLP monoclonal antibody, tezepelumab, which showed a substantial, though not statistically significant, improvement in key clinical characteristics of AD, such as the pruritus rating scale (NRS), Investigator’s Global Assessment (IGA), and reductions in both the Eczema Area and Severity Index (EASI) and the SCORing AD index (SCORAD) (122).

The interaction between myeloid immune cells and the neural system is called immune-neuro crosstalk and involves more than just TSLP (123). Sensory nerve fibers originating from the dorsal root ganglia and trigeminal ganglia innervate the skin and transmit excitatory signals (124). In healthy skin the nervous system and the immune system work together to maintain the homeostasis (125–128). Whereas in AD patients a higher density of nerve fibers around blood vessels and in the epidermis was detected in lesional skin. A change in one of the systems or an imbalance of their interaction can therefore affect both. Thus, neuropeptides and transmitters are able to initiate degranulation of mast cells resulting in an itch-scratch cycle, which is a fundamental aspect of AD pathogenesis (124, 129). A neurotransmitter that plays a crucial role in AD is the neuropeptide Y (NPY), which affects several myeloid cells like mast cells, LCs, monocytes, macrophages, and neutrophils (130, 131). Studies revealed higher levels of NPY in lesional compared to healthy skin (132, 133). Next to NPY several other neuromediators (acetylcholine, substance P, etc.) and cytokines (TSLP, Il-4, Il13, IL-33, etc.) affect the immune system (131, 134).

Although certain immune mechanisms in atopic dermatitis (AD)—such as the Th1/Th2 imbalance and the associated downregulation of type 1 immunity—are generally recognized, the detailed pathogenesis remains complex and heterogeneous (135, 136). The immune landscape of AD becomes even more complex with the identification of distinct endotypes through blood transcriptome analysis. This analysis has proposed two primary AD endotypes based on eosinophil-related expression signatures. The eosinophil-high cluster is characterized by greater dysregulation and a strong correlation between disease activity and IL-5 signaling pathways, while the eosinophil-low endotype shows minimal transcriptomic dysregulation and no significant association with disease activity (137). Expanding on this, recent research has identified four serum biomarker-based clusters (138). The first is marked by high levels of C–C chemokines and dominance of IL-1R1, the second by a mix of TH1, TH2, TH17, and epithelial-related chemokines, the third by a TH2, TH22, and pulmonary and activation-regulated chemokine (PARC) dominance, associated with more severe disease, and the fourth by a TH2 and eosinophil-low profile, which corresponds to a milder form of AD (138). In adults with moderate to severe AD, serum biomarker analysis further distinguishes between a low-inflammatory and a high-inflammatory group, with the latter showing elevated levels of TNFβ, monocyte chemoattractant protein 3 (MCP-3/CCL7), and IL-13 (139).

Ethnic differences in AD endotypes have also been identified, reflecting variations in immune responses. For instance, AD lesions in African Americans display an increased infiltration of DCs expressing the high-affinity IgE receptor (FcER1^+^), along with a skewed immune response towards TH2/TH22 and reduced TH1 and TH17 responses compared to European-American patients (140). In contrast, AD in Asian populations is characterized by a combined upregulation of TH2/TH17 responses, along with features resembling psoriasis, and a lower expression of TH1 compared to European Americans (99, 141). These findings underline the importance of considering racial differences in the management of more severe forms of AD, as these differences may influence disease presentation and treatment responses.

## Perspectives and future directions

Myeloid cells are crucial contributors to the pathophysiology of AD. Depending on their activation state, they participate in a range of functions including the initiation and maintenance of inflammation, regulation of the skin barrier, antigen presentation, and the modulation of neuroimmune interactions. The complexity and context-dependence of these responses are reflected in the growing number of therapeutic approaches targeting distinct myeloid pathways. From classical cytokine inhibition to novel small molecules and microbiome-based therapies, many of these strategies aim to interrupt key signaling pathways involving myeloid cells. The relevance of these cells as both effectors and modulators of skin inflammation makes them attractive targets for future interventions.

However, despite increasing insight into myeloid cell function in AD, several challenges remain. Current classification models often fail to capture the full phenotypic and functional heterogeneity of these cells in inflamed skin. Emerging technologies such as single-cell RNA sequencing and spatial transcriptomics have started to reveal diverse and dynamic myeloid subsets that cannot be adequately described using conventional markers. Future studies should address how these cells change over time during flares, chronic inflammation, or treatment, and how their plasticity contributes to tissue remodeling, barrier dysfunction, and itch. Translationally, strategies that reprogram rather than deplete dysfunctional myeloid populations, for example through the inhibition of key amplifiers of inflammation, may provide more targeted and sustainable therapeutic effects. In parallel, the development of cell-type-specific biomarkers could support precision medicine approaches and facilitate therapeutic monitoring.
